# Classification of breast cancer histology images using MSMV-PFENet

**DOI:** 10.1038/s41598-022-22358-y

**Published:** 2022-10-19

**Authors:** Linxian Liu, Wenxiang Feng, Cheng Chen, Manhua Liu, Yuan Qu, Jiamiao Yang

**Affiliations:** 1 School of Automation and Software Engineering, Shanxi University, Taiyuan, 030006 China; 2 School of Electronic Information and Electrical Engineering, Shanghai Jiao Tong University, Shanghai, 200240 China; 3 Institute of Marine Equipment, Shanghai Jiao Tong University, Shanghai, 200240 China; 4 Shanghai Center for Brain Science and Brain-Inspired Technology, Shanghai, 200031 China

**Keywords:** Breast cancer, Computer science, Classification and taxonomy, Image processing, Machine learning

## Abstract

Deep learning has been used extensively in histopathological image classification, but people in this field are still exploring new neural network architectures for more effective and efficient cancer diagnosis. Here, we propose multi-scale, multi-view progressive feature encoding network (MSMV-PFENet) for effective classification. With respect to the density of cell nuclei, we selected the regions potentially related to carcinogenesis at multiple scales from each view. The progressive feature encoding network then extracted the global and local features from these regions. A bidirectional long short-term memory analyzed the encoding vectors to get a category score, and finally the majority voting method integrated different views to classify the histopathological images. We tested our method on the breast cancer histology dataset from the ICIAR 2018 grand challenge. The proposed MSMV-PFENet achieved 93.0$\%$ and 94.8$\%$ accuracies at the patch and image levels, respectively. This method can potentially benefit the clinical cancer diagnosis.

## Introduction

Cancer is one of the most lethal diseases for human beings. According to the report from WHO, the number of diagnosed patients with cancer reached 19.3 million, and its casualty had increased to 10 million^1^. The gold standard for the clinical diagnosis of cancer is histopathological examination in which pathologists look into the prepared tissue sections to determine whether the tumor is benign or malignant^2^. The examination done by pathologists has two shortcomings. First, pathologists sometimes need to carefully examine many places in histological image with high magnification and large field of view. This operation is labor-intensive and time-consuming. Second, for high sensitivity and specificity, the examination demands profound professional knowledge and experience - more than 100,000 examinations often required in practice^3^. Pathologists without many practices are prone to the wrong diagnosis, causing patients to miss a valuable period of early treatment.

Benefiting from the development of image recognition technology, the computer-aided histological examination has made a breakthrough in clinical application^4–7^. It is not only improving the efficiency of examination but also reducing the rate of misdiagnosis. The computer-aided cancer diagnosis decides by analyzing the features in the image. For example, the local binary patterns, gray level co-occurrence matrix, the opponent colour local binary pattern, and many other techniques can be used to extract the features and help achieve a classification accuracy of  85$\%$^8,9^. The features extracted by these methods usually are very useful in terms of classifying a specific lesion type but are not enough for classifying multiple lesion types in one classifier.

In recent years, deep learning has achieved tremendous success in image classification^10,11^ and image enhancement^12,13^. It can extract abstract features from images with little human intervention^14,15^. Image classification methods based on deep learning frameworks, such as the convolutional neural network (CNN) and recurrent neural network (RNN), have been widely used in the pathological classification of cancer^16–22^. CNN, as a feed-forward neural network relying on its filtering and pooling layers, is good at extracting the features at a local level^23^. In contrast, RNN as a feed-backward neural network regarding the previous output as a part of its input, is good at processing the information at a global level^24^. Both frameworks have attracted the attention from the healthcare industry. Before they can be formally deployed in clinical applications, some issues must first be addressed. For example, the useful information in each input image is quite limited, due to the small image size sent to the neural networks^25,26^; however, sending the whole-slide image (WSI) into the neural network leads to a significant computational cost^27^.

There are three common ways to solve this dilemma. The first one is using a low-latency discriminator which can find out the suspicious area in the original image^28,29^. Then, a neural network can classify each case with respect to the suspicious area. The second way is segmenting the original image into multiple patches^30^. The neural network then analyzes each patch independently. In the end, the program will summarize the result of each patch in the original image and draw a conclusion about the class of this case. The third way is extracting features at multiple scales and analyzing the local and global features simultaneously^31,32^. However, using the deep learning method to select suspicious areas from the high-resolution pathological image is time-consuming. Patch-based approach^30^ will lose feature context information if the relationship between patches cannot be well solved. In addition, the methods based on multi-scale^31,32^ fail to provide targeted models for multi-scale feature representation.

Furthermore, the breast cancer datasets such as BACH^33^ and BreakHis^8^ often have a limited volume. Using the limited data to train the network can easily lead to over-fitting and become useless in the application^34^. Transfer learning^35^ and data augmentation^36^ can somehow solve this problem. In the transfer learning, the network model is pre-trained on a generalized dataset and then trained on the specific dataset where your application is defined. In the image augmentation, the dataset is effectively expanded by various affine transformations^37^. This method is especially effective for the large-scale pathological images.

Here, we propose multi-scale, multi-view progressive feature encoding network (MSMV-PFENet). To extract more effective suspicious region, the key-region-extraction module (KREM) selects the image patches from the original pathological images, according to the density of cell nuclei. The progressive feature encoding network (PFENet), as our multi-scale feature representation model, progressively extracts features from the images and encodes the features into vectors. The feature discrimination network (FDNet) integrates encoding vectors of multiple-scales through bidirectional long short-term memory (BiLSTM)), mining potential feature relationships between local and global, to avoid loss of information between patches. We combine the advantages of CNN and RNN to preserve the short-term and long-term spatial correlation between the features at different scales. In addition, the majority-voting method summarizes the results of six views to determine the final category, avoiding one-sidedness under a single view. Testing the model on the breast cancer histology (BACH) dataset^33^ and Yan’s dataset^30^, MSMV-PFENet can achieve a good performance in terms of accuracy, precision, recall, and F1 score.

## Methods

**Figure 1:**
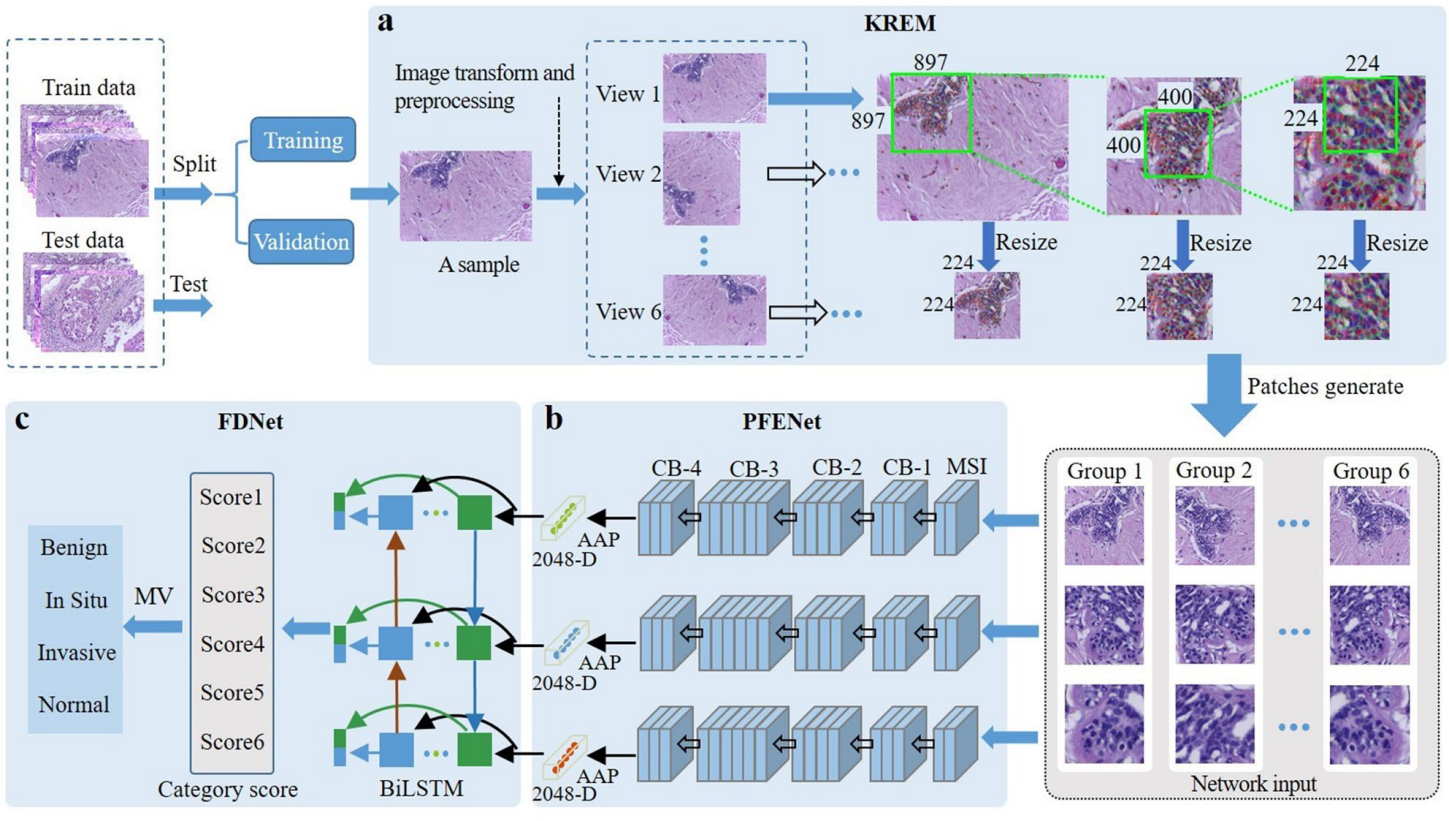
Diagram of breast cancer pathological image classification using MSMV-PFENet. (**a**) KREM. (**b**) PFENet. (**c**) FDNet. MSI: multi-scale input; PIL: preprocessing input layer; CB: convolution block; AAP: adaptive average pooling; D: dimensions; MV: majority vote.

The proposed MVMS-PFENet took up the high-resolution HE staining pathological images (2048 $\times$ 1536) and would classify the images into four categories—normal, benign, carcinoma in situ, and invasive carcinoma. As shown in Fig. 1, we performed six view transformations (raw, 90$^\circ$, 180$^\circ$, 270$^\circ$, *x*-flip, *y*-flip) on an input image after preprocessing. In each view, the key regions at three scales were selected from the region with a high density of cell nuclei (Fig. 1a), we got six groups of multi-scale key image patches. The PFENet encoded each group of patches into vectors (Fig. 1b), then a two-layer BiLSTM fused the encoding vectors of the patches at different scales to get category scores in each view (Fig. 1c). Finally, the FDNet analyzed the scores obtained from the six views and determined the type of cancer according to majority voting (Fig. 1c). The detailed structure of MVMS-PFENet is shown in Table 1.

**Table 1:** The architecture of MSMV-PFENet. “Conv” layer shown in the table corresponds to the sequence BN-ReLU-convolution (BN, batch normalization; ReLU, rectified linear unit); FC: fully connected layer.

Block	Layers	Output size (C $\times$ H $\times$ W)	Parameters	Repetition
MSI	Input-1	3 $\times$ 224 $\times$ 224	Downscale 4$\times$	–
	Input-2	3 $\times$ 224 $\times$ 224	Downscale 1.79$\times$	–
	Input-3	3$\times$ 224 $\times$ 224	Downscale 1$\times$	–
PIL	Conv	64 $\times$ 112 $\times$ 112	7 $\times$ 7, Stride 1	–
	Maxpool	64 $\times$ 56 $\times$ 56	3 $\times$ 3, Stride 1	–
CB-1	Conv	64 $\times$ 56 $\times$ 56	1 $\times$ 1, Stride 1	$\times$ 3
	Conv	64 $\times$ 56 $\times$ 56	3 $\times$ 3, Stride 1	
	Conv	256 $\times$ 56 $\times$ 56	1 $\times$ 1, Stride 1	
CB-2	Conv	128 $\times$ 56 $\times$ 56	1 $\times$ 1, Stride 1	$\times$ 4
	Conv	128 $\times$ 28 $\times$ 28	3 $\times$ 3, Stride 2 or 1	
	Conv	512 $\times$ 28 $\times$ 28	1 $\times$ 1, Stride 1	
CB-3	Conv	256 $\times$ 28 $\times$ 28	1 $\times$ 1, Stride 1	$\times$ 6
	Conv	256 $\times$ 14 $\times$ 14	3 $\times$ 3, Stride 2 or 1	
	Conv	1024$\times$ 14 $\times$ 14	1$\times$1, Stride 1	
CB-4	Conv	512 $\times$ 14 $\times$ 14	1 $\times$ 1, Stride 1	$\times$ 3
	Conv	512 $\times$ 7 $\times$ 7	3 $\times$ 3, Stride 2 or 1	
	Conv	2048 $\times$ 7 $\times$ 7	1 $\times$ 1, Stride 1	
AAP	AdaptiveAvgPool2d	2048	–	–
	Concatenation	2048 $\times$ 3	–	–
FD	BiLSTM	256	1	$\times$ 4
Output	FC	4	–	–
	Softmax	4	–	–

In KREM, inspired by the standard histological examination, we created a module to extract the suspicious areas in a large original image for further analysis to reduce computational cost. The designed module selected the image patches at different scales from the original image with respect to the density of cell nuclei due to its underlying relation with the lesion.

**Figure 2:**
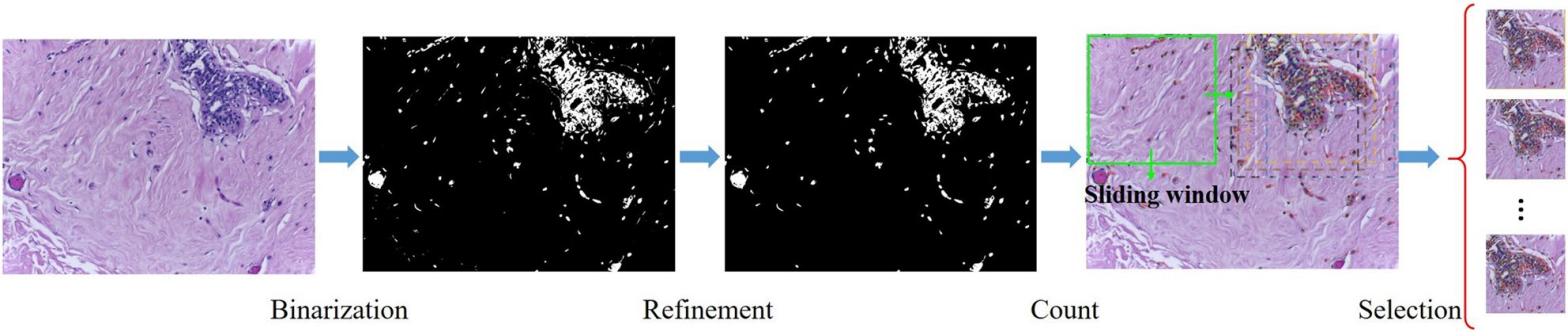
Diagram showing how the KREM extracts image patches. The extraction process includes four key steps: binarization, refinement, count, and selection.

As shown in Fig. 2, the KREM selection process consists of four key steps: Eq. (1) We first converted the colorful input image to a grayscale image and set a threshold according to the mean of pixel values. As a result, we obtained a binary image in which the white pixels represented the area occupied by the nuclei. Equation (2) Via the statistical analysis of all the histological images at 200$\times$ magnification in the dataset, we found that the majority of nuclei were in an oval shape with an area of more than 80 pixels and the ratio of area to circumference was less than 2.5. According to this criterion, irrelevant regions were removed to refine the region of the nucleus. Equation (3) We used a sliding window to search in the image and calculated the number of detected nuclei in the window. Equation (4) The image patches with the highest number of nuclei would be selected. Within a selected region, a smaller sliding window repeated the previous process to select the image patches at the next scale. In this paper, we used sliding windows at three scales. The sizes of sliding windows at the three scales were 897 $\times$ 897, 400 $\times$ 400, and 224 $\times$ 224, respectively. Their corresponding step sizes respectively were 80, 40 and 20 pixels.

**Figure 3:**
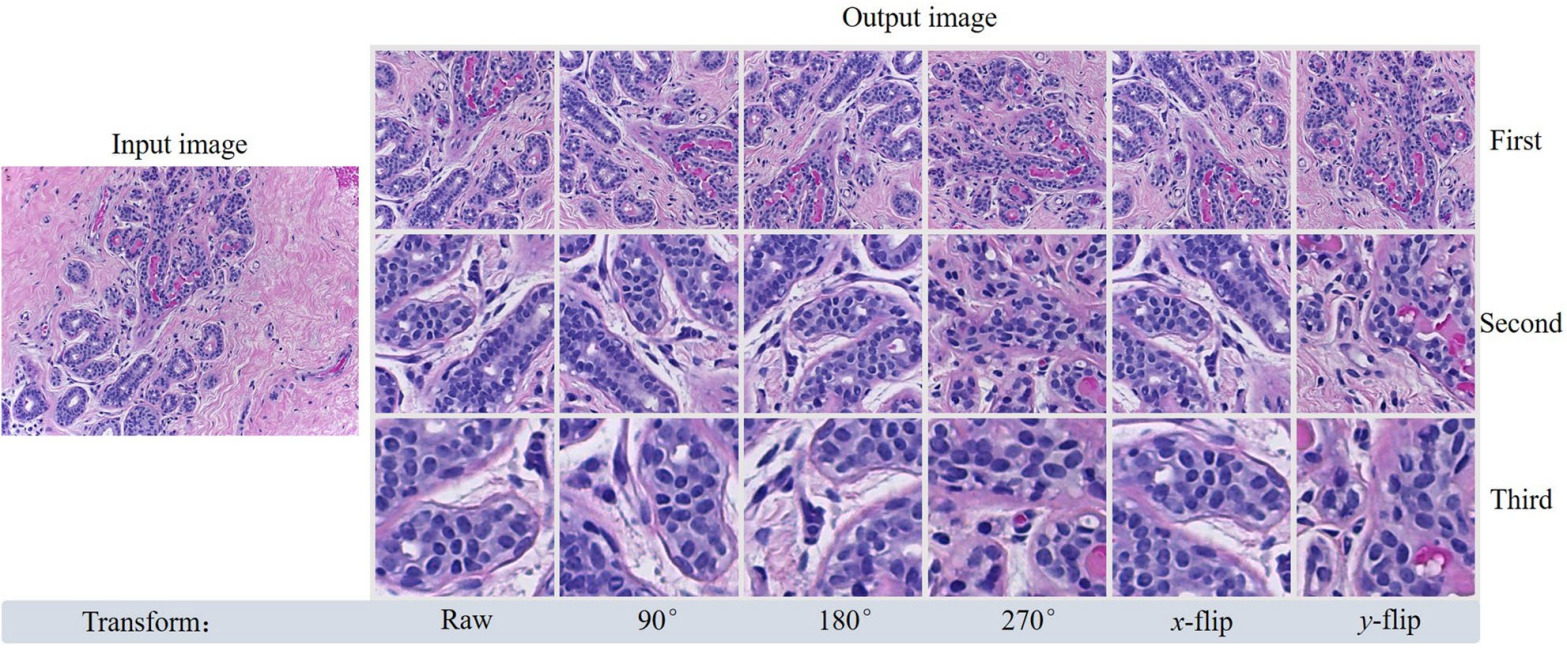
An example showing the image patches selected by the KREM. The left is the input image, and the right is the densest area of cell nuclei on three scales selected from image after performing six view transformations. First, second and third are the scale levels.

In the training phase, KREM first rotated each pathological image by 90$^\circ$, 180$^\circ$, 270$^\circ$ , and inverted relative to the *x*- and *y*-axes to augment the limited dataset. Then we picked out 12 regions with the densest nuclei from candidate windows at the first selecting level to further expand the amount of data. Unlike other data augmentation methods, this step is based on the data augmentation achieved by our proposed sliding window nuclei density detection technique. This can reduce invalid patches and preserve the information of the lesions. After that, our pathological image dataset was amplified by 72 times. In the testing phase, KREM performed six view transformations on a testing image, and only picked the densest region of cell nuclei in each view. We observe from 6 views, thereby avoiding the sliding step length too long and could not cover the area with dense nuclei. Figure 3 shows the image patches selected by the KREM.

**Figure 4:**
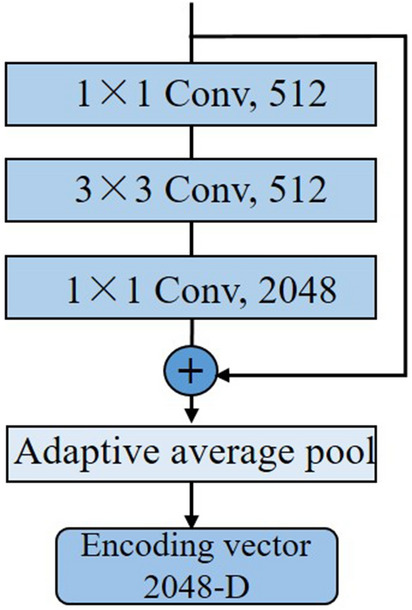
Diagram of obtaining the encoding vector. The adaptive average pooling layer flattens the output feature map into a 2048-dimensional (D) encoding vector to represent the key region image patch.

The structure of PFENet was composed of three parallel networks. The PFENet represented the image patches at multiple scales by vectors to facilitate the fusion of features of different scales (Fig. 4). Before inputting the network, we used bilinear interpolation to subsample the image patches at the ratios of 4$\times$, 1.79$\times$, and 1$\times$. The three image patches produced in each view were used as a group, corresponding to the three inputs of the PFENet. The PFENet at all scales shares the same structure similar to ResNet50^38^. We did not adopt a network structure larger than ResNet50 to limit the computation cost in the feature extraction. The PFENet progressively extracted low-level color, texture, and deep semantic features layer by layer through convolution to learn pathological information at various scales. Finally, three 2048-dimensional encoding vectors were obtained via the adaptive average pooling layer. This would reduce the amount of calculation in the BiLSTM to get category scores.

**Figure 5:**
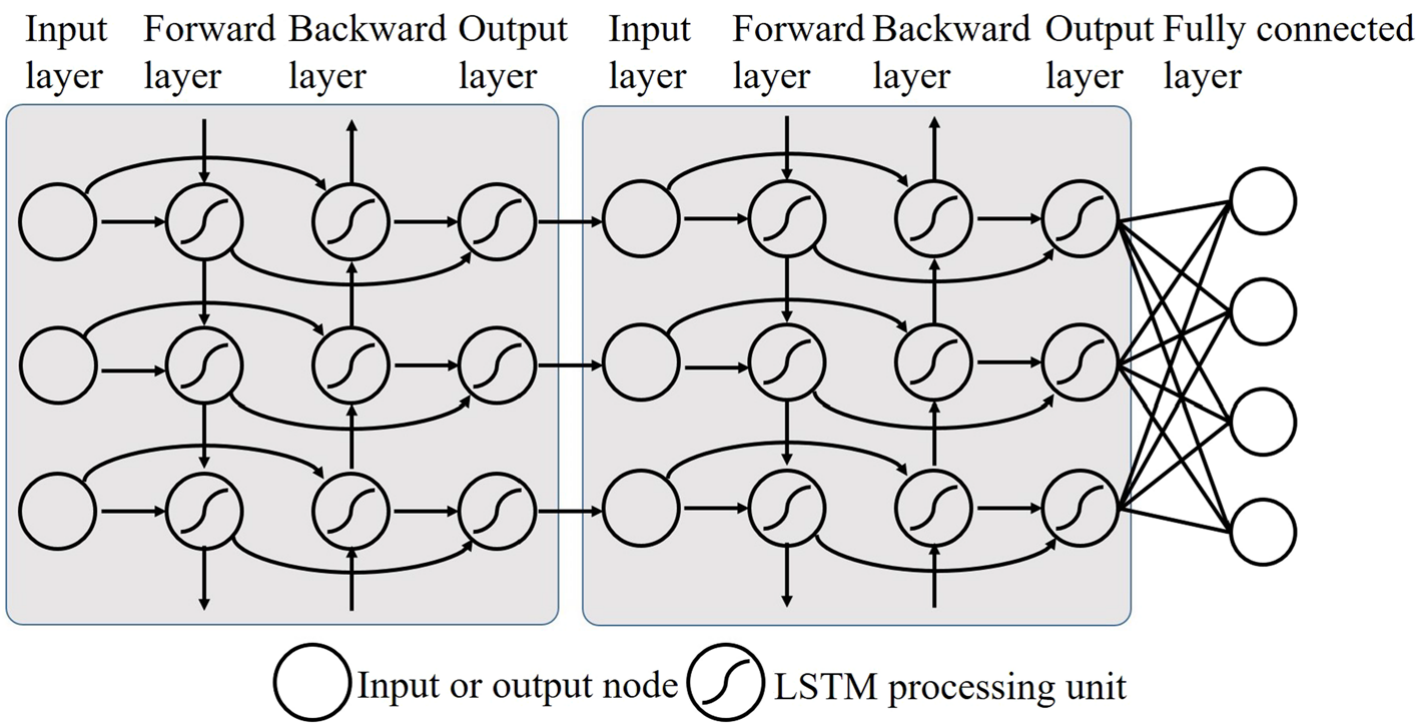
Diagram of two-layer BiLSTM we used. Each circle represents a node, the input layer has three nodes and the output layer has four nodes. The direction arrow indicates the direction of information flow.

The encoding vectors at different scales represent global and local features, and they have strong correlations at one view. We used two layers of BiLSTM to share and learn the feature representation. The two-layer BiLSTM is a part of FDNet, as shown in Fig. 1c, the input is three consecutive sequences corresponding to the output of PFENet. BiLSTM is composed of two LSTM (Fig. 5). A directed ring is formed between LSTM units, which creates the internal state of the network and makes information flow between units. The output of LSTM depends on the previous input and the current internal state. The encoding vectors were fed into the network, and the information flowed in three channels to establish a global and local relationship, thereby making up for the limitations of features at different scales. In this way, BiLSTM fused multi-scale pathological features and mined the potential feature relationships between input sequences, and then got pathological image category scores in each view. Finally, we got scores on six views. To realize the end-to-end classification, PFENet and BiLSTM were jointly trained.

**Figure 6:**
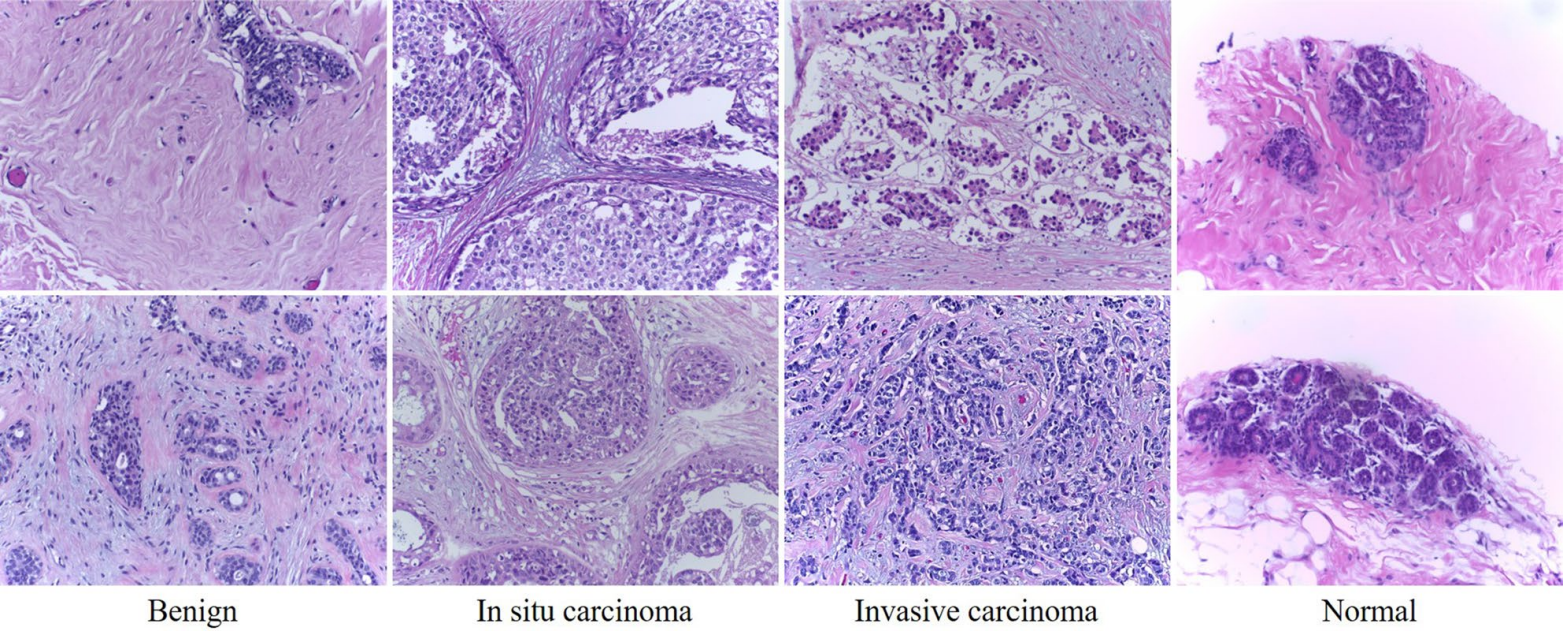
Microscopic H &E images of four types of tumors from the BACH dataset (200$\times$ magnification).

Pathological images are special in that they have no directional specification. The diagnosis results should not be affected by the view and should be consistent. However, images clipped from the WSI may have blank areas, as shown in the last column of Fig. 6. The existence of the blank areas will cause inaccurate extraction of image patches in key areas. In addition, the window has a stride in sliding and cannot traverse the image completely, which will also affect the acquisition of key areas. Therefore we need to further discriminate categories from different views. The final category is determined by the results of six views using the majority voting method.

## Results

### Experimental setup

We trained and tested the MSMV-PFENet on a platform equipped with one NVIDIA Tesla V100 GPU (32G memory) and 24-core Intel Xeon Platinum 8168 Processor (33M Cache, 2.70 GHz). We trained the network with a batch size of 64 through 27,000 iterations, using stochastic gradient descent (SGD) in which we set the momentum to 0.9 and weight decay to 1e-4. To accelerate the training, we set the initial learning rate at 0.01 for the first 30 batches and then divided the rate by 10 after every 10 batches, until the rate was equal to 1e-4. The BACH dataset^33^ used in our experiment contained 400 training images and 100 testing images (Fig. 6, Table 2). The dimensions of images are 2048 $\times$ 1536 pixels, and the pixel size is $0.42\,\upmu \mathrm{m} \times 0.42\,\upmu \mathrm{m}$. We divided training images into two sets with a ratio equal to 4:1 for training and validation.

**Table 2:** Information about the BACH dataset^33^.

BACH dataset	Normal	Benign	In situ	Invasive	Total
Train and validate	100	100	100	100	400
Test	25	25	25	25	100

To avoid over-fitting due to the small size of the dataset, we used transfer learning^35^ and data augmentation^36^ techniques. PFENet originated from ResNet50 pre-trained from ImageNet. PFENet did not have the average pooling layers and fully connected layers that were essential for ResNet50 and replaced by an adaptive average pooling layer. We randomly initialized the parameters in FDNet and trained FDNet with PFENet together. PFENet encoded the features in the image into a vector, and FDNet took the vector in for classification. We fine-tuned the whole model according to the classification feedback so that FDNet could discriminate the various features and images correctly. The specific details of dataset augmentation are presented in the Methods section. We also normalized the color in the images to avoid the inconsistency caused by different staining protocols^39^.

To demonstrate the classification capabilities of MSMV-PFENet, we used the same image-level and patch-level as in^30,31^ to evaluate the network from multiple aspects. We defined the accuracy (*Acc*), precision (*Pre*), recall (*Rec*), and F1 score (*F*1) as

1 $$Acc=\frac{TP+TN}{TP+TN+FP+FN}, $$

2 $$Pre=\frac{TP}{TP+FP}, $$

3 $$Rec=\frac{TP}{TP+FN}, $$

4 $$F1=\frac{2 \times Pre \times Rec}{Pre+Rec}, $$

Here, *TP*, *TN*, *FP*, and *FN* are the true-positive, true-negative, false-positive, and false-negative, respectively. These criteria had also been adopted elsewhere^40,41^.

### Comparison with other methods

To show the classification capability of MSMV-PFENet, we compared the accuracy given by our network with that in the other published^30,39,42–44^. Our MSMV-PFENet could achieve an accuracy of 93.0$\%$ at patch-level (Table 3); the value was higher than those reported elsewhere. The comparison illustrated that a well-trained MSMV-PFENet could effectively extract the most important features in the original images at both the local and global levels. Moreover, the BiLSTM and majority voting mechanism in our network further boosted the accuracy through comprehensive consideration of local and global features. As a result, MSMV-PFENet achieved an accuracy of 94.8$\%$ at image-level.

**Table 3:** Reported classification accuracy at patch-level and image-level using various networks.

Method	Patch (%)	Image (%)
Araújo et al.^42^	66.7	77.8
Kohl et al.^39^	–	83.0
Rakhlin et al.^43^	–	87.2
Golatkar et al.^44^	79.0	85.0
Yan et al.^30^	82.1	91.3
Our work	93.0	94.8

### Results on the key regions extraction

To check the influence of various methods extracting the image patches from the original images, we compared our method guided by the density of cell nuclei with other methods of randomly selecting patches. When we chose the random selection, training MSMV-PFENet was difficult, because not all of the lesions in the original image would be sent to the classifier for analysis, which led to relatively low accuracy ( 85.4$\%$). In comparison, our method made the training process much easier than the random selection method and benefited the classification power of MSMV-PFENet, based on the fact that cancer cells were often associated with increased nuclear size, irregular nuclear contours, and disturbed chromatin distribution. At the patch-level, the accuracy could reach  93.0$\%$.

### Results on different network combinations

To find an optimal design for feature encoding and discriminating in MSMV-PFENet, we carried out a comprehensive study comparing various architectures (Table 4). For feature encoding, we chose a fully connected layer as the feature discriminator and tested FENet, PFENet, VGG16^26^, GoogLeNet^45^, and ResNet-101^38^ as the feature encoder (Table 4, rows 1–6). PFENet in this test provided the best accuracy at both the patch and image levels among the five architectures because the PFENet containing 3 CNNs in parallel could encode the features from the local and global levels simultaneously. The global features represented at low resolution also reduced the computational cost and made the classifier more efficient than analyzing a high-resolution image as a whole.

**Table 4:** Performance comparison of various network architecture combinations.

Feature encoding method	Discrimination method	Patch ($\%$)	Image ($\%$)
Single FENet	FC	84.3	85.4
Double FENet	FC	86.6	88.9
VGG16	FC	78.8	80.1
GoogLeNet	FC	82.0	82.6
ResNet-101	FC	85.5	86.1
PFENet	FC	88.7	92.5
PFENet	SVM	89.1	90.0
PFENet	BiLSTM	89.5	92.3
PFENet (best)	BiLSTM $\times$ 2	93.0	94.8
PFENet	BiLSTM $\times$ 3	91.2	93.7

After we settled down the issue with feature encoding, we tested several candidates, including the FC, support vector machine (SVM) and BiLSTM, for the classifier (Table 4, rows 6–10). BiLSTM outperformed the other two candidates, because it could effectively associate the information at the local and global levels. Unfortunately, training BiLSTM was difficult. The deeper the BiLSTM network was, the harder the network converged to a qualified discriminator. Balancing the tradeoff between the accuracy and feasibility of training, we finally chose a network design with a double-layer BiLSTM for the following demonstrations.

### Results of the whole network

**Figure 7:**
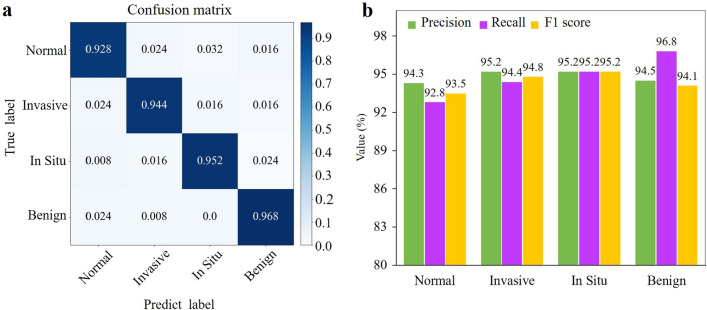
Results of the whole network on the BACH test dataset. (**a**) Confusion matrix illustrating the classification done by MSMV-PFENet. (**b**) Histogram of the precision, recall and F1 score for the evaluation of MSMV-PFENet.

Figure 7a shows the confusion matrix calculated in our test on the BACH test dataset. The results confirmed that our MSMV-PFENet could accurately associate the histological images with their clinical outcomes, including normal, benign, in situ carcinoma, and invasive carcinoma. The precision, recall, and F1 score as additional references were also calculated, and these scores had always been higher than 92.8$\%$ (Fig. 7b). We noticed that the misclassification rate was higher for the normal tissue, which perhaps was induced by the subtle inconsistency in the tissue morphology among the populations with different genotypes.

**Figure 8:**
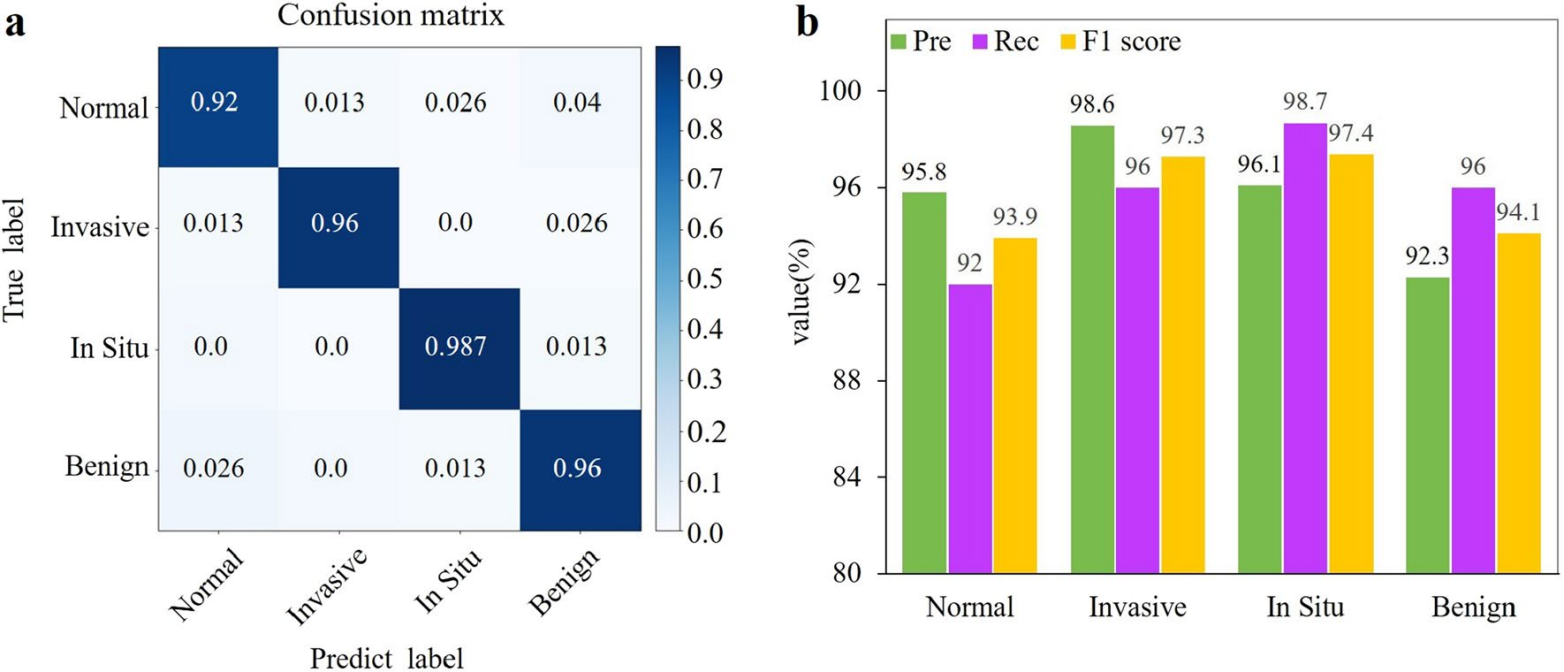
Results of the whole network on the Yan’s dataset. (**a**) Confusion matrix illustrating the classification done by MSMV-PFENet. (**b**) Histogram of the precision, recall and F1 score for the evaluation of MSMV-PFENet.

In addition, we also verified our method on the Yan’s dataset^30^. This dataset contains 3771 diversified high-resolution pathological images. Figure 8a shows the confusion matrix calculated on the test dataset. The precision, recall, and F1 score were still maintained at higher than 92$\%$ (Fig. 8b), and the accuracy reached 95.6$\%$. The results demonstrated that MVMS-PFENet also can achieved good performance on a larger dataset.

## Conclusion

In this paper, we proposed MSMV-PFENet to classify the high-resolution breast histopathological images. We first selected the image patches at multiple scales from the high-resolution images, using a sliding window and the statistics of cell nuclei, so as to avoid selecting invalid image patches and preserve the data information of lesions. The PFENet then extracted the color, texture and deep semantic features of pathological images and converted these image patches into the encoding vectors to improve the efficiency of multi-scale feature processing. The BiLSTM fused the encoding vectors and mined the potential feature between sequences to get category scores in each view. Transfer learning accelerated the convergence of PFENet as well as BiLSTM and improved the generality of the model. The image augmentation expanded the dataset through various rotations and flips and reduced the likelihood of over-fitting. The majority voting method combined the category scores from six views (original, 90$^\circ$ rotated, 180$^\circ$ rotated, 270$^\circ$ rotated, x-flip, y-flip) and further improved the accuracy of classification. The results were verified on the BACH dataset, and the accuracy of patch-level and image-level reached 93$\%$ and 94.8$\%$ respectively. We also verified our method on the dataset released by Yan et al.^30^, and achieved the state-of-the-art results. Our method therefore could be a potentially useful tool for pathologists working on cancer.

## Data Availability

The data that support the findings of this study are available from the corresponding author upon reasonable request.
